# TIME PERSPECTIVE OF THE PERSONALITY OF PATIENTS WHO HAD SEVERE AND MEDIUM COVID-19

**DOI:** 10.1192/j.eurpsy.2023.1666

**Published:** 2023-07-19

**Authors:** E. R. Semenova, E. Pervichko, A. Kulikova, I. Shishkova, J. Konyukhovskaya

**Affiliations:** 1 Moscow State University named after ‘M. V. Lomonosov’, Moscow; 2 Ryazan State Medical University, Ryazan, Russian Federation

## Abstract

**Introduction:**

In connection with the COVID-19 pandemic and its consequences, the question of how personal time constructs are modified becomes highly relevant. In particular, it is important to understand the specifics of the personality’s time perspective in patients who have undergone COVID-19 in varying degrees of severity.

**Objectives:**

To study the time perspective of personality in patients who have undergone COVID-19 in medium and severe forms.

**Methods:**

The study was conducted from February to April 2022. The first group of the sample (the medium form with hospitalization) consisted of 52 respondents (46.2% - men, 53.8% - women, M age =31.2 years; S=6.7). The second group (severe form with hospitalization) consisted of 48 patients (60% - men and 40% - women, M age =33.0 years; S=7.8). We used: “The questionnaire of the time perspective of the personality of F. Zimbardo (Short version)”; (Zimbardo, Boyd, 1997; Syrtsova, 2008), the “Scale of Time experience” questionnaire (Golovakha, Kronik, 2008) and the descriptive analysis.

**Results:**

Respondents with a medium form of the disease have more developed hedonistic present (3.2±0.6), future (3.8±0.4) and positive past (3.7±0.6). The negative past (2.6±0.7) and fatalistic present (2.4±0.6) are the least represented in their lives. Respondents with a severe form have a more developed negative past (3.4±0.7), hedonistic present (3.4±0.4), future (3.7±0.5) and positive past (3.6±0.7). The fatalistic present is the least represented in their life (3.0±0.5). When analyzing the factors of time experiencing, it was revealed that respondents with a medium form perceive time as moderately continuous (3.4± 0.8), moderately tense (3.4± 0.8) due to pronounced emptiness and compactness, and also treat time on average not very positively (3.2± 1.0). Respondents with a severe form also perceive time as moderately continuous (3.4± 0.7), rather tense (4.1±1.1) due to pronounced saturation, compactness and rapidity, while they treat time on average moderately positively (2.5± 0.9).

**Conclusions:**

The time perspective in patients with medium form is characterized by planning and achieving future goals, and these respondents also show a fairly high degree of acceptance of their own past, in which any experience is an experience that contributes to development and led to today’s state. In respondents with a severe form, along with normative scores on the positive past scale, there is also an increase in negative perception of the past, which is reflected in an increased degree of rejection of their own past, causing disgust, full of pain and frustration, as well as a hedonistic, risky attitude to time and life, while an orientation towards pleasure, excitement, excitement, enjoyment in the present and lack of concern for future consequences or sacrifices in favor of future rewards. Assistance and help to such respondents should be in the focus of specialists of the relevant profile.

**Disclosure of Interest:**

None Declared

